# The AP-1 Binding Sites Located in the *pol* Gene Intragenic Regulatory Region of HIV-1 Are Important for Viral Replication

**DOI:** 10.1371/journal.pone.0019084

**Published:** 2011-04-19

**Authors:** Laurence Colin, Nathalie Vandenhoudt, Stéphane de Walque, Benoît Van Driessche, Anna Bergamaschi, Valérie Martinelli, Thomas Cherrier, Caroline Vanhulle, Allan Guiguen, Annie David, Arsène Burny, Georges Herbein, Gianfranco Pancino, Olivier Rohr, Carine Van Lint

**Affiliations:** 1 Laboratoire de Virologie Moléculaire, Institut de Biologie et de Médecine Moléculaires (IBMM), Université Libre de Bruxelles (ULB), Gosselies, Belgium; 2 Institut Pasteur, Unité de Régulation des Infections Rétrovirales, Paris, France; 3 IUT Louis Pasteur de Schiltigheim, University of Strasbourg, Schiltigheim, France; 4 Department of Virology, EA3186, IFR133, Franche-Comte University, Hôpital Saint-Jacques, Besançon, France; Institut Pasteur Korea, Korea

## Abstract

Our laboratory has previously identified an important intragenic region in the human immunodeficiency virus type 1 (HIV-1) genome, whose complete functional unit is composed of the 5103 fragment, the DNaseI-hypersensitive site HS7 and the 5105 fragment. These fragments (5103 and 5105) both exhibit a phorbol 12-myristate 13-acetate (PMA)-inducible enhancer activity on the herpes simplex virus thymidine kinase promoter. Here, we characterized the three previously identified AP-1 binding sites of fragment 5103 by showing the PMA-inducible *in vitro* binding and *in vivo* recruitment of c-Fos, JunB and JunD to this fragment located at the end of the *pol* gene. Functional analyses demonstrated that the intragenic AP-1 binding sites are fully responsible for the PMA-dependent enhancer activity of fragment 5103. Moreover, infection of T-lymphoid Jurkat and promonocytic U937 cells with wild-type and mutant viruses demonstrated that mutations of the intragenic AP-1 sites individually or in combination altered HIV-1 replication. Importantly, mutations of the three intragenic AP-1 sites led to a decreased *in vivo* recruitment of RNA polymerase II to the viral promoter, strongly supporting that the deleterious effect of these mutations on viral replication occurs, at least partly, at the transcriptional level. Single-round infections of monocyte-derived macrophages confirmed the importance of intragenic AP-1 sites for HIV-1 infectivity.

## Introduction

Human immunodeficiency virus type 1 (HIV-1) gene expression is regulated at the transcriptional level by *cis*-acting elements located in the viral 5′ long terminal repeat (5′LTR) and leader region, by *trans*-acting factors including the viral transactivating Tat protein and cellular transcription factors that are either constitutively expressed in most cells (such as Sp1 and Oct-1) or inducible in T cells and macrophages (such as NF-κB and NFAT), and by the chromatin organization of the HIV-1 provirus [1]. In addition to the 5′LTR enhancer, our laboratory has previously identified a phorbol 12-myristate 13-acetate (PMA)-inducible intragenic enhancer located in the coding region of HIV-1 [2]. It is composed of two functional domains termed fragment 5103 (located at the end of the *pol* gene and encompassing nucleotides (nt) 4079 to 4342, where nt +1 is the beginning of U3 in the 5′LTR) and fragment 5105 (encompassing nt 4781 to 6026, which correspond to *vif* and the first coding exon of *tat*). These fragments both exhibit a PMA-inducible enhancer activity on the herpes simplex virus (HSV) thymidine kinase (TK) promoter in the human epithelial HeLa cell line, but no significant activity in T-lymphoid and monocyte-macrophage cell lines [2].

**Figure 1:**
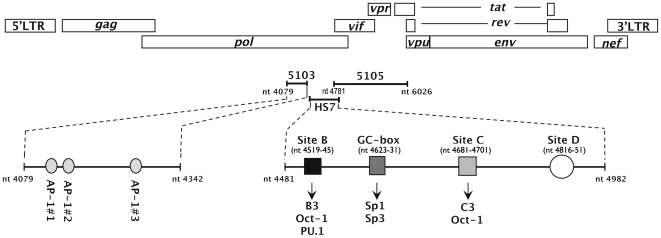
Schematic representation of the intragenic *cis*-regulatory region of HIV-1. The complete functional unit of the intragenic *cis*-regulatory region encompasses nt 4079 to nt 6026 and is composed of the 5103 fragment, the hypersensitive site HS7 and the 5105 fragment. The three AP-1 binding sites of fragment 5103 are indicated as well as the previously characterized binding sites of the HS7 region (site B which binds Oct-1 and a T cell specific complex termed B3 or the monocyte/macrophage lineage-specific complex PU.1, the GC-box which binds Sp1 and Sp3, site C which binds Oct-1 and a T-cell specific complex termed C3 and site D which binds unidentified complexes [6]).

Furthermore, our laboratory has studied the chromatin organization of HIV-1 proviruses integrated in several latently-infected cell line models of T-lymhoid (ACH2 and 8E5) or monocytic (U1) origin [3], [4], [5]. Besides the anticipated presence of DNaseI-hypersensitive sites in the two LTRs, a single major hypersensitive site (named HS7) was identified in the part of the *pol* gene coding for the integrase (centred around nt 4490–4766) [3], [5], thereby indicating a potential transcriptional regulatory role of this region. This constitutive hypersensitive site was observed only in a cell line of monocytic origin (U1) and not in two cell lines of lymphoid origin (8E5 and ACH2) [3], suggesting a certain cellular specificity associated to this site. Interestingly, the HS7 is positioned between the previously identified 5103 and 5105 fragments. Several ubiquitous and cell-specific transcription factors have been shown to be recruited in the HS7 region (including Oct-1, Sp1/Sp3 and PU.1) [5], [6] and to be important for viral infectivity [6]. Altogether, these results demonstrate the importance of the intragenic *cis*-regulatory region, whose complete functional unit is composed of the 5103 fragment, the hypersensitive site HS7 and the 5105 fragment (see Figure 1).

Given that the enhancer activity of the HIV-1 intragenic region had been demonstrated to be inducible by phorbol-esters [2], our laboratory has previously examined this region for the presence of binding sites for PMA-inducible transcription factors (such as NF-κB, AP-1, AP-2 and AP-4). In this context, we have identified three AP-1 binding sites in fragment 5103 by *in silico* analyses based on nucleotide sequence homologies to the consensus DNA recognition motif of AP-1 transcription factors [5′-(A/T)T(G/T)(A/C)(G/C)TCA(G/C/A)-3′] [7]. Short oligonucleotides containing the two first AP-1 sites or the third AP-1 site were demonstrated to bind *in vitro* affinity-purified AP-1/c-Jun or AP-1 present in PMA-induced HeLa nuclear extracts [7]. In addition to the two AP-1 binding sites previously described in the 5′LTR negative regulatory element (NRE) of different HIV-1 neurotropic strains [8], three AP-1 sites have been characterized by our laboratory downstream of the transcription start site in a large nucleosome-free region termed HS4 (nt 465-720), which functions as an enhancer towards HIV-1 5′LTR transcriptional activity [9].

The AP-1 transcription factors, originally identified by their binding to the enhancer element of the simian virus 40 (SV40) promoter [10], function as homo- or heterodimers composed of members of the *jun*, *fos* and *atf* multigene families [11]. Dimerizing via their basic leucine zipper domain and thereby members of the wider B-ZIP family, AP-1 transcription factors bind DNA at palindromic sequences, also known as 12-*O*-Tetradecanoylphorbol-13-acetate (TPA or PMA) Responsive Elements (TREs) [12], to regulate both the constitutive and the inducible transcription of a wide variety of cellular and viral genes [11], [13]. The negative or positive role of AP-1 in the transcriptional activity of a specific target gene depends on cell type, dimer-composition, abundance of each dimer partner, translational regulation mechanisms [14], post-translational modifications [including phosphorylation [15], [16] and sumoylation [17], [18]] and AP-1 interactions with transcriptional (co)factors such as SWI/SNF [19]. Particularly, phosphorylation of AP-1 by the protein kinase ERK1/2 (Extracellular Regulated Kinase 1/2) promotes AP-1 interaction with NF-κB in the HIV-1 5′LTR, leading to transcriptional activation [20], [21]. AP-1 activity is induced by a wide range of stimuli such as growth factors, cytokines, bacterial and viral infections and numerous physical and chemical stresses. AP-1 plays important roles in various cellular processes including cellular proliferation and differentiation, apoptosis, signalling, stress responses, cell migration and tumorigenesis [11].

In this report, we have characterized biochemically each of the three intragenic AP-1 binding sites. We have examined the functional role of these AP-1 binding sites (individually or in combination) for the PMA-dependent enhancer activity of the 5103 fragment, in the presence or absence of the viral transactivating Tat protein. Importantly, we have investigated the biological significance of the intragenic AP-1 binding sites for HIV-1 replication both in cell lines and in primary macrophages (MDMs).

## Materials and Methods

### Cell lines and cell culture

The T-lymphoid cell lines Jurkat [22] and SupT1 [23] were obtained from the AIDS Research and Reference Reagent Programme (National Institute of Allergy and Infectious Diseases [NIAID], National Institutes of Health [NIH]). The monocytic cell line U937 was obtained from the American Type Culture Collection (Manassas, VA). The U937, Jurkat and SupT1 cell lines were maintained in RPMI 1640-Glutamax I medium (Invitrogen) supplemented with 10% fetal bovine serum (FBS), 50 µg/ml streptomycin and 50 units/ml penicillin. The adherent cell lines HeLa (a human epithelial cell line derived from a cervical carcinoma and transformed by human papilloma virus type 18), TZM-bl (an HeLa-derivative cell line that expresses high levels of HIV-1 receptor CD4 and both co-receptors CXCR4 and CCR5, and contains β-galactosidase and luciferase reporter genes under the control of the HIV-1 LTR) and 293T (a human embryonic kidney cell line) were cultured in Dulbecco's modified Eagle's-Glutamax I medium (Invitrogen) supplemented with 5% FBS, 50 µg/ml streptomycin and 50 units/ml penicillin (containing 1 mM sodium pyruvate for the 293T cells). All cells were grown at 37°C in an atmosphere of 5% CO_2_.

### MDMs isolation and culture

Peripheral blood mononuclear cells (PBMCs) were isolated from buffy coats of healthy seronegative donors (Centre de Transfusion Sanguine Ile-de-France Rungis, Paris, France) by density centrifugation on a Ficoll-Hypaque gradient (PAA). Monocytes were isolated from PBMCs by plastic adherence and differentiated into macrophages by culturing for 7 to 11 days in MDM medium (RPMI 1640 medium supplemented with 200 mM L-glutamine, 100 units/ml penicillin, 100 µg/ml streptomycin, 10 mM HEPES, 10 mM sodium pyruvate, 50 µM β-mercaptoethanol, 1% minimum essential medium vitamins, and 1% nonessential amino acids) containing 15% of human AB serum (PAA) in hydrophobic Teflon dishes (LumoxTM, D Dutscher). Prior to infection experiments, MDMs were harvested, washed and resuspended in MDM medium containing 10% fetal calf serum.

### Plasmid constructs and generation of mutated reporter constructs by site-directed mutagenesis of the *pol* gene AP-1 binding sites

The A-Fos dominant negative construct was kindly provided by Dr. Charles Vinson (NCI, National Cancer Institute, Bethesda, MD 20892, USA) [24]. The expression vectors coding either for the one-exon form of Tat (72 amino acids, named pTat72) or the two-exon form of Tat (101 amino acids, named pTat101) were previously described [25]. The pTK reporter construct contains the luciferase gene under the control of the HSV TK minimal promoter and was generated by subcloning the XmaCI-XhoI fragment from the pGL2-TK (see [26]) into the XmaCI-XhoI-restricted pGL3-basic vector (Promega). The pLTR containing the HIV-1 5′LTR upstream of the luciferase gene in the context of the pGL3-basic vector was previously described [6].

Mutations of the AP-1 binding sites were introduced in the 5103 fragment following the QuikChange site-directed mutagenesis kit manufacturer's protocol (Stratagene), using 50 ng of the pCV10 construct as a substrate (pBluescript II SK vector which contains an ApaI-EcoRI fragment corresponding to nt 2011-5743 of the HIV-1_NL4.3_ genome and previously described [6]) and the following pairs of mutated oligonucleotide primers (mutations are highlighted in boldface and the AP-1 motifs are underlined on the coding strand): CV1364-CV1365 (site AP-1#1mut: FW: 5′-GCACAACCAGATAAG**TCA** GAATCAGAGT TAGTCAGTCAA-3′; RV: 5′-TTGACTGACTAACTCTGATTCTGACTTATCTGGT TGTGC-3′), CV1366-CV1367 (site AP-1#2mut, FW: 5′-AAGAGTGAATCAGAGTT **G**GT**T**AGTCAAATAATAGAG-3′; RV: 5′-CTCTATTATTTGACTAACCAACTCTGAT TCACTCTT-3′), CV728-CV729 (site AP-1#3mut, FW: 5′-CAAGTAGATAAATTGG T**T**AGTGCTGGAATC-3′; RV: 5′-GATTCCAGCACTAACCAATTTATCT ACTTG-3′) and CV1151-CV1152 (site AP-1#1+2mut, FW: 5′-ACAACCAGATAAG**TCA** GAATCA GAGTT**G**GT**T**AGTCAAATAATAG-3′; RV:5′-CTATTATTTGACTAACCAACTCTGATT CTGACTTATCTGGTTGT-3′). Following PCR, the samples were treated for two hours with the endonuclease DpnI, which is specific for methylated and hemi-methylated DNA (target sequence 5′-Gm^6^ATC-3′) and digested the parental wild-type DNA template to select for mutation-containing plasmids. Mutated clones were fully resequenced between ApaI and EcoRI restriction sites after identification (Genomex). The four mutated resulting pCV10-derivative plasmids were designated pCV1208 (AP-1#1mut), pCV1209 (AP-1#2mut), pCV1202 (AP-1#3mut) and pCV1210 (AP-1#1+2mut), respectively. A pCV10-derivative construct containing a combination of the three AP-1 mutations described above was also generated and designated pCV887 (AP-1#totmut). The ApaI-EcoRI mutagenized fragments from pCV887, pCV1202, pCV1208, pCV1209 and pCV1210 were introduced into the unique ApaI-EcoRI sites of the two-LTRs-containing infectious HIV-1 molecular clone pNL4.3 (reagent no. 114, received from the AIDS Research and Reference Reagent Program, NIAID, NIH) to generate pCV1352 (termed pHIV-1-AP-1#totmut), pCV1393 (pHIV-1-AP-1#3mut), pCV1394 (pHIV-1-AP-1#1mut), pCV1395 (pHIV-1-AP-1#2mut) and pCV1396 (pHIV-1-AP-1#1+2mut), respectively. As a control, a nonmutated ApaI-EcoRI fragment was purified from pCV10 and cloned in an identical manner into the unique ApaI-EcoRI sites of the pNL4.3, construction now referred to as pHIV-1.

The *pol* gene fragment (nt 4079-4342) corresponding to the 5103 fragment from the infectious proviral molecular clone pNL4-3 was amplified by PCR. XmaCI sites were introduced into the PCR primers, and the XmaCI-restricted PCR fragment was cloned into the unique XmaCI site of the pTK, placing the amplified fragment upstream of the TK-luciferase transcriptional unit in the sense or antisense orientation, thereby generating the pTK-5103s-wt or the pTK-5103as-wt, respectively. The 5′ oligonucleotide primer encompassed the coding strand sequence from nt 4079 to 4099 and contained an added XmaCI restriction site (underlined) at the 5′ end (CV596: 5′-TCCCCCGGGATCC[4079]AGATAAGAGTGAA TCAGAGTT-3′). The 3′ oligonucleotide primer encompassed the complementary sequence from nt 4328 to 4342 and contained an added XmaCI site (underlined) at the 5′ end (CV598: 5′-TCCCCCGGGATCCCAGCTG [4342]GCTACTATTTCTTTT-3′). Fragments containing the 5103 region mutated in the three AP-1 binding sites individually or in combination were PCR amplified from the pCV887, pCV1202, pCV1208, pCV1209 and pCV1210 plasmids (see above). The 5′ and 3′ oligonucleotide primers were as described above except for the pCV887, pCV1208 and pCV1210 plasmids, which contain mutations in the AP-1#1 site. In these latter cases, the 5′ oligonucleotide primer was as follows: 5′-TCCCCCGGGATCC[4079]AGATAAG**TCA**GAATCAGAGTT-3′ (the added XmaCI restriction site is underlined and mutations in the AP1#1 site are highlighted in boldface). The different amplified fragments were digested with XmaCI and then ligated into the unique XmaCI site of pTK in the antisense orientation, thereby generating the pTK-5103as-AP-1#totmut, the pTK-5103as-AP-1#3mut, the pTK-5103as-AP-1#1mut, the pTK-5103as-AP-1#2mut and the pTK-5103as-AP-1#1+2mut constructs, respectively.

### Electrophoretic Mobility Shift Assays (EMSAs)

**Figure 2:**
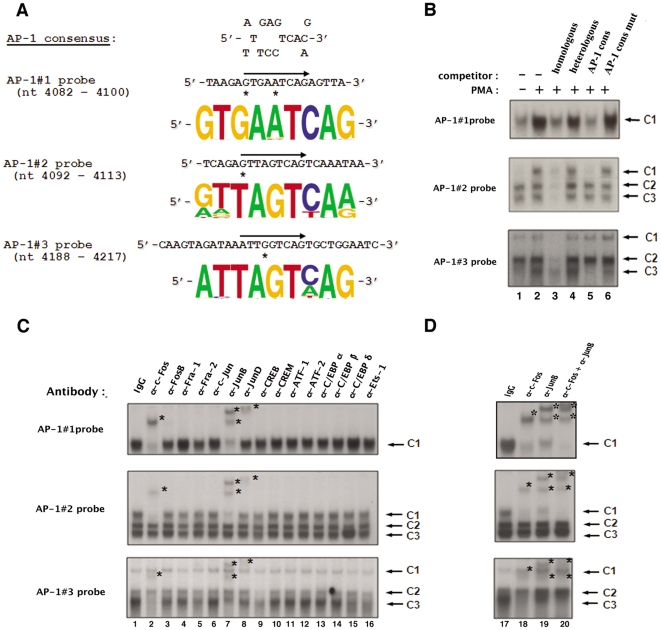
AP-1 transcription factors specifically bind *in vitro* to each of the three intragenic AP-1 sites of fragment 5103. (**A**) The nucleotide sequence of the wild-type AP-1#1, AP-1#2 and AP-1#3 site oligonucleotides used as probes in our EMSAs are shown properly aligned with the AP-1 consensus sequence. The position of the AP-1 binding site is indicated by an arrow on each coding strand and mismatches in the AP-1 sites with respect to the AP-1 consensus sequence are designated by asterisks. The conservation of the intragenic AP-1 sites was assessed by comparing their sequences at the nucleotide level based on the full spectrum of HIV and SIV sequences compiled in the HIV compendium database (hiv-web.lanl.gov). Sequence logos that represent the frequency of the nucleotide present at each position in the intragenic AP-1 binding sites were generated based on these sequence analyses for each intragenic AP-1 site. (**B**) The AP-1#1, AP-1#2 and AP-1#3 oligonucleotide probes were incubated with nuclear extracts (10 µg) from mock-treated (lane 1) or PMA-treated (lanes 2 to 6) HeLa cells in the absence of competitor (lanes 1 and 2) or in the presence of a molar excess (5 fold) of a competitor corresponding to the homologous AP-1 site (lane 3), to the heterologous Sp1 consensus (lane 4; nucleotide sequence of the coding strand: 5′-ATTCGATCGGGGCGGGGCGAGC-3′), to the AP-1 consensus (lane 5; nucleotide sequence of the coding strand: 5′-CGCTTGATGACTCAGCCGGAA-3′) or to the mutated AP-1 consensus (lane 6; nucleotide sequence of the coding strand: 5′-CGCTTGATGACT**TG**GCCGGAA-3′, where mutations compared to the consensus are indicated in bold). The figure shows the specific retarded bands of interest, which are indicated by arrows. The terms C1, C2 and C3 refer to complexes 1, 2 and 3. (**C**) Nuclear extracts from PMA-treated HeLa cells (10 µg) were incubated, before the addition of the AP-1 probe, either with a purified rabbit IgG as a negative control (lane 1), or with an antibody directed against AP-1 family members including c-Fos (lane 2), FosB (lane 3), Fra-1 (lane 4), Fra-2 (lane 5), c-Jun (lane 6), JunB (lane 7) and JunD (lane 8), or with an antibody directed against other members of the B-ZIP family such as CREB (lane 9), CREM (lane 10), ATF-1 (lane 11), ATF-2 (lane 12), C/EBPα (lane 13), C/EBPβ (lane 14) and C/EBPδ (lane 15), or with an antibody directed against Ets-1 (lane 16). The figure shows the specific retarded bands of interest indicated by arrows. Supershifted complexes are indicated by asterisks. (**D**) Ten µg of nuclear extracts from PMA-treated HeLa cells were incubated with an antibody directed against c-Fos (lane 18), an antibody directed against JunB (lane 19) or a combination of both antibodies (lane 20). A purified rabbit IgG was used as a negative control (lane 17). The AP-1#1, AP-1#2 or AP-1#3 oligonucleotide probe was then added to the mixture. The figure shows the specific retarded bands of interest indicated by arrows. The supershifted complexes are indicated by asterisks.

**Figure 3:**
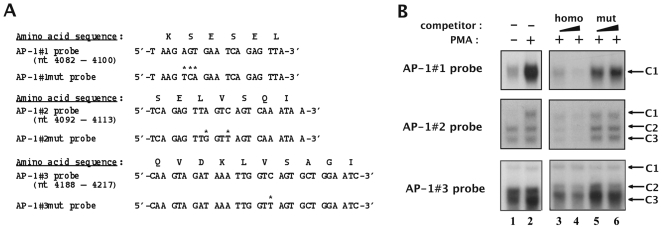
Identification of point mutations which abolish AP-1 transcription factors' *in vitro* binding to their respective binding sites located in fragment 5103. (**A**) The wild-type and mutated AP-1#1, AP-1#2 and AP-1#3 oligonucleotide sequences are indicated as well as the corresponding underlying amino acid sequence of the viral reverse transcriptase. Base pairs which were modified in the mutated versions of the AP-1 binding sites relative to the wild-type versions are indicated by asterisks. (**B**) The AP-1#1, AP-1#2 and AP-1#3 oligonucleotide probes were incubated with nuclear extracts (10 µg) from mock-treated (lane 1) or PMA-treated (lanes 2 to 6) HeLa cells in the absence of competitor (lanes 1 and 2) or in the presence of increasing molar excesses (2 and 4 fold) of each respective homologous AP-1 oligonucleotide (lanes 3 and 4) or of the corresponding mutated AP-1 oligonucleotide (lanes 5 and 6). The figure shows the specific retarded bands of interest, which are indicated by arrows.

Nuclear extracts were prepared from cells using a rapid protocol described by Osborn et al. [27]. All buffers contained the following protease inhibitors: antipain (10 µg/ml), aprotinin (2 µg/ml), chymostatin (10 µg/ml), leupeptin (1 µg/ml) and pepstatin (1 µg/ml). Protein concentrations were determined according to the Bradford methodology [28], with bovine serum albumin (BSA) as a standard. The DNA sequences of the coding strand of the wild-type and mutated versions of the AP-1#1, AP-1#2 and AP-1#3 probes used in this study are listed in Figures 2A and 3A, respectively. The various lengths of these oligonucleotides, which results from design constraints, may account for the different numbers of complexes observed with the different probes. EMSAs were performed as described previously [26]. Briefly, nuclear extracts (10 µg of protein) were first incubated for 10 min in the absence of probe and specific competitor DNA in a 16 µl reaction mixture containing 10 µg of DNase-free BSA (Amersham Biosciences), from 0.5 to 2 µg of poly(dI-dC) (Amersham Biosciences) as non-specific competitor DNA, 50 µM ZnCl_2_, 0.25 mM DTT, 20 mM HEPES (pH 7.3), 60 mM KCl, 1 mM MgCl_2_, 0.1 mM EDTA and 10% (v/v) glycerol. 30,000 cpm of probe (80 - 100 fmol) were then added to the mixture with or without a molar excess of an unlabeled specific DNA competitor, and the mixture incubated 20 min at room temperature. Samples were subjected to electrophoresis on 6% polyacrylamide gels at 120 V for 2-3 h in 1x TGE buffer [25 mM Tris-acetate (pH 8.3), 190 mM glycine and 1 mM EDTA]. Gels were dried and autoradiographed for 24-48 h at -80°C. For supershift assays, antibodies against c-Fos (sc-52X), FosB (sc-48X), Fra-1 (sc-605X), Fra-2 (sc-171X), c-Jun (sc-44X), JunB (sc-73X), JunD (sc-74X), CREB (sc-240X), CREM (sc-440X), ATF-1 (sc-243X), ATF-2 (sc-242X), C/EBPα (sc-9314X), C/EBPβ (sc-150X), C/EBPδ (sc-151X) and Ets-1 (sc-350X) or a purified rabbit immunoglobulin (IgG; sc-2027) were added to the reaction mixture and incubated for 30 min on ice before the addition of the radiolabelled probe.

### Transient transfection and luciferase reporter assays

HeLa cells were transiently transfected using JetPEI™ (POLYplus) according to the manufacturer's protocol. Briefly, one day before transfection, cells were seeded at a density of 8×10^3^ cells/well in 96-well plates. For each well, 200 ng of DNA were diluted into 25 µl of 150 mM NaCl. The transfection reagent JetPEI™ (0.45 µl/well) was diluted into 150 mM NaCl (25 µl/well). An aliquot of 25 µl of this JetPEI™/NaCl solution was added to the 25 µl DNA solution, and the JetPEI™/NaCl/DNA mixture incubated for 15 min at room temperature before being added dropwise to each well. All transfection mixtures contained the pRL-TK (in which a cDNA encoding the *Renilla* luciferase is under the control of the HSV TK promoter region) as an internal control for transfection efficiency. At 24 hours after transfection, cells were mock-treated or treated with PMA (20 nM) (Sigma). At 24 post-treatment, transfected cells were lysed and assayed for luciferase activity. *Firefly* luciferase activities derived from the HSV TK promoter were normalized with respect to the *Renilla* luciferase activities by using the DualGlo-luciferase reporter assay system (Promega), and to protein concentrations using the Bradford quantification method [28]. Statistical analyses of the data were performed and p-values are indicated in the figure legends.

### Chromatin immunoprecipitation assays

The chromatin immunoprecipitation (ChIP) assays were performed as previously described by Flanagin *et al.* [29], [30] with minor modifications. Briefly, cells in exponential growth phase were cross-linked for 10 min at room temperature with 1% formaldehyde (whose action was then neutralized with TRIS-Glycin 12.5 mM), washed twice with phosphate-buffered saline (PBS) and lysed in IP buffer (150 mM NaCl, 50 mM Tris–HCl pH 7.5, 5 mM EDTA, 0.5% NP-40, 1% Triton X-100) containing protease inhibitors (Roche). To detect chromosomal flanking regions, pellets were sonicated (Bioruptor sonicator) to obtain DNA fragments of an average size of 400 nt. Chromatin immunoprecipitations were performed in commercially available protein-A-coated 96 well plates (Pierce). After two washes (200 µl PBS/well), well walls were blocked with 200 µl blocking buffer (IP buffer completed with 5% BSA and 100 µg/ml sheared salmon sperm DNA) for 30 min. Wells were cleared and then incubated with antibodies (0.5 µg) in 100 µl blocking buffer/well for 60 min. Wells were cleared and chromatin samples (80,000 cells in 100 µl blocking buffer) were added and incubated overnight at 4°C. Wells were then washed three times with 200 µl IP buffer and once with 200 µl TE buffer, before being incubated with 100 µl elution buffer (25 mM Tris base, 1 mM EDTA, pH 9.8, 200 µg/ml proteinase K) for 15 min at 55°C, followed by 30 min at 75°C. DNA samples were stored (−20°C) in the same Matrix ChIP plates for repeated use. Antibodies directed against AP-1 family members used in this study were described above, an antibody directed against RNA polymerase II (sc-899) was also used, and a purified IgG (I-1000, Vector Laboratories) was used as a control for immunoprecipitation to test aspecific binding to the plate. Quantitative real-time PCR (qPCR) reactions were performed using the MesaGreen qPCR mastermix (Eurogentec) with 5 µl of the eluted DNA product. Relative quantification using the standard curve method was performed for each primer pair and 96-well Optical Reaction plates were read in an Applied Biosystems AbiPrism 7300 real-time PCR instrument (Absolute Quantification Method). Fold enrichments were calculated as percentages of input values. Primer sequences used for quantification in the 5103 fragment region (FW: 5′- GGGAATCATTCAAGCACAACC-3′; RV: 5′- TCTTGGGCCTTATC TATTCCATC-3′), in the nuc-1 region ([31]; FW: 5′- GACTGGTGAGTACGCCAAA -3′; RV: 5′- TAATACTC GACGCTCTCGC -3′) and in an unrelated viral region corresponding to the *vpr* gene (FW: 5′- GCAACAACTGCTGTTTATCCATT-3′; RV: 5′- TTTCTTGCTCTCCTCTGTCGAG-3′) were designed using the software Primer Express 2.0 (Applied Biosystems).

### Production of viral stocks

The generated wild-type and mutated full-length molecular clones were used to produce stocks of wild-type and mutant viruses (termed HIV-1, HIV-1-AP-1#totmut, HIV-1-AP-1#1mut, HIV-1-AP-1#2mut, HIV-1-AP-1#3mut and HIV-1-AP-1#1+2mut). The infectious DNAs (750 ng) were transiently transfected into 10^7^ Jurkat cells using the DEAE-dextran procedure. At 24 h posttransfection, cultures were co-cultivated with 10^7^ SupT1 cells to allow rapid and efficient recovery of progeny viruses. HIV-1 stocks were prepared from culture supernatants after filtration through a 0.45-µm-pore-size membrane (Nalgene) and were quantified by determining p24 antigen concentration using an enzyme-linked immunosorbent assay (ELISA) (Innogenetics). Each viral stock was verified by sequence analyses of HIV-1 genomic RNA in order to confirm the presence of the originally introduced mutations using the following procedure: viral particles from each stock were pelleted by ultracentrifugation (at 20,000× *g* for 2 h at 4°C) and digested with RNase-free DNase I (110 U/ml for 15 min at room temperature [Invitrogen]) in the presence of RNaseOUT (40 U/ml; Invitrogen) to remove contaminating DNA. HIV-1 genomic RNA was purified using the High Pure Viral RNA Kit (Roche Applied Science) following the manufacturer's protocol. cDNA synthesis was performed by the Titan One Tube RT-PCR Kit method (Roche Applied Science). cDNAs were then amplified by PCR with a 5′ oligonucleotide primer corresponding to nt 3882–3907 (5′-GCAGCCAATAGG GAAACTAAATTAGG-3′) and a 3′ oligonucleotide primer corresponding to nt 5056–5035 (5′-GCCATCTGTTTTCCATAATCCC-3′). PCR fragments were subcloned into the vector pCR4 Blunt-TOPO (Zero Blunt TOPO PCR Cloning Kit [Invitrogen]). After identification of recombinant clones, three inserts from each construct were sequenced (Genomex). The nucleotide sequences of all three clones were identical and confirmed the presence of originally introduced mutations.

### Infection assays in cell lines

First, infectivity of wild-type and mutant viruses was assessed in TZM-bl cells. In brief, exponentially growing cells were seeded at 6×10^3^ cells/200 µl in 96 well plates and were infected or not with equal amounts of wild-type or mutant viruses (corresponding to 20 ng of p24 antigen). At 72 h post-infection, TZM-bl cells were lysed in PBS and luciferase activity was measured (Promega). Second, infections of Jurkat and U937 cells were performed by incubating 0.5×10^6^ cells with 50 ng of p24 protein of wild-type or mutant viruses (at 37°C for 2 h in 500 µl of culture medium). After infection, cells were pelleted at 300× *g*, washed three times with 1 ml of culture medium, resuspended in 1 ml of culture medium, and grown under standard conditions. Every 2 or 3 days, aliquots of 200 µl were removed from the infected cultures and replaced by fresh medium. The aliquots were assayed for p24 concentration in triplicate in order to monitor the kinetics of viral replication. In addition, total RNA was extracted from infected cells at days 5, 10 and 15 post-infection using the RNeasy Plus Mini Kit (Qiagen) and digested with TURBO DNase I (Ambion) to ensure the removal of genomic DNA. First strand cDNA was synthesized using SuperScript III Reverse Transcriptase and random primers (Invitrogen). qPCR reactions were performed as described above with the comparative C_t_ (ΔΔC_t_) quantification method. The following sets of primers were used for amplification of initiated viral transcripts (TAR: FW: 5′-GTTAGACCAGATCTGAGCCT-3′; RV: 5′-GTGGGTTCCCTAG TTAGCCA-3′) and elongated transcripts (Tat mRNAs: FW: 5′- ACTCGACAGAGGAGAGCA AG-3′; RV: 5′- GAGTCTGACTGTTCTGATGA-3′), using β-actin (FW: 5′- GTCGACAACGGCT CCGGC-3′; RV: 5′- GGTGTGGTGCCAGATTTTCT-3′) to normalize the results.

### Production of VSV-G pseudotyped viruses and single-round infectivity assays in MDMs

HIV-1/VSV-G (Vesicular Stomatitis Virus Glycoprotein) pseudotypes were produced by transiently cotransfecting 293T cells with the generated HIV-1_NL4.3_ wild-type or mutant full length molecular clones and the pMD2 VSV-G expression vector. Supernatants containing pseudotyped viral particles were collected at 48 h post-transfection, filtered through a 0.45-µm-pore-size membrane and stored at −80°C. Viral stocks were termed HIV-1_VSV-G_, HIV-1-AP-1#1mut_VSV-G_, HIV-1-AP-1#2mut_VSV-G,_ HIV-1-AP-1#3mut_VSV-G,_ HIV-1-AP-1#1+2mut_VSV-G_ and HIV-1-AP-1#totmut_VSV-G_. HIV-1 p24 antigen in viral stocks was quantified using an ELISA kit (Innogenetics). Each viral stock was verified by sequence analyses of HIV-1 genomic RNA in order to confirm the presence of the originally introduced mutations as described above. For single-round infections, MDMs (0.8×10^5^ cells/well in 96 well plates) were infected in triplicate with 100 µl of HIV-1/VSV-G pseudotyped viruses (containing equivalent concentrations of p24) using a spinoculation protocol (1 h centrifugation at room temperature at 1,200× g followed by 1 h incubation at 37°C). Cells were then washed with PBS and cultured in MDM medium. At days 3 and 6 post-infection, the supernatant of each well was replaced by fresh medium and at day 10 post-infection, p24 concentration in the supernatants was measured by ELISA.

### Western blotting

HIV-1 lysates were prepared by ultracentrifugation of each virus stock (500 ng of p24 at 20,000× *g* for 2 h at 4°C) and the pellets were resuspended in Laemmli buffer at a concentration of 8.3 ng of p24/µl. Lysates were heated at 95°C for 5 min, separated by electrophoresis on a 10% polyacrylamide gel and transferred onto a polyvinylidene difluoride membrane. The membrane was then blocked in Tris-buffered saline (TBS) containing 5% non-fat dry milk and incubated with a purified human anti-HIV-1 IgG (NIH AIDS Research and Reagent Program, reagent no. 192 donated by Dr Alfred Prince). A horseradish peroxidase-conjugated goat anti-human IgG (Pierce) was then used for enhanced chemiluminescence detection (Cell Signalling). The dominant negative A-Fos mutant was detected in nuclear extracts from transiently transfected cells using an anti-FLAG antibody (Sigma) followed by a second horseradish peroxidase-conjugated anti-mouse IgG used for enhanced chemiluminescence detection (Cell Signalling).

## Results

### AP-1 transcription factors specifically interact *in vitro* with each of the three AP-1 sites located in the HIV-1 5103 fragment

Our laboratory has previously identified three AP-1 binding sites in the 5103 fragment of the HIV-1 intragenic *cis*-regulatory region by *in silico* analyses [7]. Short oligonucleotides containing the two first AP-1 binding sites or the third AP-1 binding site, respectively, were previously demonstrated by our laboratory to bind *in vitro* affinity-purified AP-1/c-Jun or AP-1 present in PMA-treated HeLa nuclear extracts by EMSAs (competition experiments) [7]. In order to pursue the *in vitro* characterization of the intragenic AP-1 sites, we performed additional EMSAs using three oligonucleotide probes containing each site individually (termed AP-1#1, AP-1#2 and AP-1#3 probe, respectively, and designed based on the nucleotide sequence of the infectious HIV-1_NL4.3_ isolate). The three intragenic AP-1 binding sites are very well conserved at the nucleotide level as represented in Figure 2A by sequence logos that symbolize the frequency of the nucleotide present at each position in these binding sites based on sequence analyses of the full spectrum of HIV and SIV (Simian Immunodeficiency Virus) sequences compiled in the HIV compendium database (hiv-web.lanl.gov). Of note, the third AP-1 binding site sequence is slightly different in the HIV-1_NL4.3_ strain compared to the other strains since it contains a guanine residue at position 4 in the TRE sequence instead of an adenine or a cytosine (as in the AP-1#3 site from most of other HIV-1 strains or in the AP-1 consensus sequence), a mutation that has been shown in the literature to impede AP-1 binding to TREs [32].

The radiolabelled probes were incubated with nuclear extracts from mock-treated (Figure 2B, lane 1) or PMA-treated (20 nM for one hour; lanes 2 to 6) HeLa cells in the absence (lanes 1 and 2) or presence of different unlabelled double-stranded oligonucleotides as competitors, corresponding either to the homologous sequence (i.e. the same sequence as that of the radiolabelled probe; lane 3), or to the heterologous Sp1 binding site consensus (lane 4), or to the AP-1 binding site consensus (lane 5; named AP-1 cons), or to a mutated version of the AP-1 binding site consensus (lane 6; named AP-1 cons mut). As shown in Figure 2B, incubation of the AP-1#1 probe with nuclear extracts from mock-treated HeLa cells resulted in the formation of a broad retarded complex designated AP-1#1-C1 (Fig. 2B, top panel, lane 1). When nuclear extracts from PMA-treated HeLa cells were used, the intensity of the AP-1#1-C1 complex increased markedly (Fig. 2B, top panel, lane 2). Moreover, formation of the AP-1#1-C1 complex was competed for by a molar excess of the unlabeled homologous AP-1#1 oligonucleotide (Fig. 2B, top panel, lane 3), but not by the same molar excess of a heterologous oligonucleotide of unrelated sequence (Fig. 2B, top panel, lane 4), thereby demonstrating the sequence specificity of complex AP-1#1-C1 binding to the AP-1#1 probe. Furthermore, the addition of a molar excess of an unlabeled oligonucleotide corresponding to an AP-1 binding site consensus (Fig. 2B, top panel, lane 5) hindered the formation of the AP-1#1-C1 complex, whereas this complex formation was not competed for by the same molar excess of a mutated version of the AP-1 consensus (Fig. 2B, top panel, lane 6), thereby demonstrating that the AP-1#1-C1 complex is specific to the AP-1 motif.

Incubation of the AP-1#2 probe with nuclear extracts from mock-treated HeLa cells resulted in the formation of three retarded complexes designated AP-1#2-C1 to AP-1#2-C3 (Fig. 2B, middle panel, lane 1) that were all specific to the AP-1#2 sequence as demonstrated by competition with homologous and heterologous oligonucleotides (Fig. 2B, middle panel, lanes 3 and 4). The slower migrating complex AP-1#2-C1 was strongly induced following PMA-treatment of the cells (Fig. 2B, middle panel, lane 2). A molar excess of the unlabelled AP-1 consensus oligonucleotide (Fig. 2B, middle panel, lane 5), but not of its mutated version (Fig. 2B, middle panel, lane 6), specifically inhibited the formation of this PMA-inducible AP-1#2-C1 complex, whereas no differences in the binding of the AP-1#2-C2 and AP-1#2-C3 complexes were observed, thereby showing that the AP-1#2-C1 complex is specific to the AP-1 motif.

Finally, three retarded complexes were observed using the AP-1#3 probe, including a PMA -inducible complex termed AP-1#3-C3 (Fig. 2B, bottom panel, lanes 1 and 2). These three retarded complexes were shown to be specific to the AP-1#3 sequence by competition experiments with homologous and heterologous competitors (see Fig. 2B, bottom panel, lanes 3 and 4, respectively). Moreover, the AP-1#3-C3 complex was demonstrated to be specific to the AP-1 motif since its formation was completely inhibited by a molar excess of the AP-1 consensus oligonucleotide (Fig. 2B, bottom panel, lane 5), but only slightly affected by the same molar excess of the mutated AP-1 consensus oligonucleotide (lane 6). However, even with a low concentration of poly(dI-dC), a non-specific DNA competitor, the AP-1#3-C3 complex was difficult to observe. These low affinity binding properties are in good correlation with the presence of a guanine residue at position 4 in the TRE of the AP-1#3 site in the HIV-1_NL4-3_ isolate, which markedly decreases AP-1 binding efficiency [32].

In order to directly identify the factors present in the PMA-inducible AP-1#1-C1, AP-1#2-C1 and AP-1#3-C3 complexes, we performed supershift assays using antibodies directed against individual members of the AP-1 family and against members of the related C/EBP family of B-ZIP transcription factors (Figures 2C and 2D). Radiolabelled AP-1 probes incubated with PMA-treated HeLa nuclear extracts and either a purified rabbit IgG (Fig. 2C, lane 1) as a negative control or an antibody directed against c-Fos, FosB, Fra-1, Fra-2, c-Jun, JunB, JunD, CREB, CREM, ATF-1, ATF-2, C/EBPα, C/EBPβ, C/EBPδ or Ets-1 (Fig. 2C, lanes 2 to 16).

The addition of an antibody directed against JunD or against c-Fos interfered with the formation of complex AP-1#1-C1 and led to the appearance of a supershifted complex of decreased mobility (Fig. 2C, top panel, lane 8; Fig. 2C, top panel, lane 2 and Fig. 2D, top panel, lane 18, respectively). The addition of an anti-JunB antibody also hindered the formation of complex AP-1#1-C1, generating two supershifted complexes (Fig. 2C, top panel, lane 7; Fig. 2D, top panel, lane 19). The AP-1#1-C1 complex intensity decreased less markedly following the addition of an antibody directed against JunD than following the addition of an anti-cFos or an anti-JunB antibody (Fig. 2C, top panel; compare lane 8 with lanes 2 and 7), suggesting a smaller contribution of JunD in the composition of the AP-1#1-C1 complex. Interestingly, when both anti-cFos and anti-JunB antibodies were included in the same binding reaction, the AP-1#1-C1 complex entirely disappeared (Fig. 2D, top panel, lane 20), confirming that c-Fos and JunB corresponded to the predominant AP-1 species which bound to the AP-1#1 site. In contrast, the binding pattern observed following incubation of the AP-1#1 probe with PMA-treated HeLa nuclear extracts was weakly affected or not affected at all by the addition of antibodies directed either against the other AP-1 family members (Fig. 2C, top panel, lanes 3 to 6), or against other members of the B-ZIP family (Fig. 2C, top panel; lanes 9 to 15) or against the Ets-1 transcription factor (Fig. 2C, top panel; lane 16), supporting the notion that the PMA-inducible retarded complex AP-1#1-C1 did not contain these other proteins. As a control, all the antibodies that did not supershift AP-1 complexes in this study were demonstrated to be functional in EMSAs with other radiolabelled probes (data not shown). Moreover, no retarded band was affected neither by the addition of a purified rabbit IgG used as a negative control (Fig. 2C, top panel, lane 1; Fig. 2D, top panel, lane 17) nor by the addition of antibodies to the probe alone (data not shown), indicating the specificity of the protein-antibody interactions. Similar results were obtained for the AP-1#2-C1 and AP-1#3-C3 complexes (Fig. 2C and 2D, middle and bottom panels, respectively).

Taken together, our results demonstrate that each of the three intragenic AP-1 sites located in the *pol* gene 5103 fragment bind *in vitro* the c-Fos, JunB and to a lesser extent JunD transcription factors of the AP-1 family.

### Identification of mutations abolishing AP-1 binding to the AP-1 sites of fragment 5103

To further characterize biochemically and functionally the AP-1 sites located in fragment 5103, we designed point mutations aimed at abolishing AP-1 binding to their respective binding site, without altering the underlying amino acid sequence of the viral reverse transcriptase.

We substituted the adenine residue at position 4086, the guanine residue at position 4087 and the thymine residue at position 4088 from the AP-1#1 probe by a thymine, a cytosine and an adenine residue, respectively (see Figure 3A, top panel) to generate its mutated version designated AP-1#1mut. We evaluated the impact of this 3-bp mutation on AP-1 binding by competition EMSAs with the AP-1#1 oligonucleotide as a probe and nuclear extracts from mock-treated and PMA-treated HeLa cells (Fig. 3B, top panel, lane 1 and lanes 2 to 6, respectively). As expected, the PMA-inducible AP-1#1-C1 specific complex was inhibited by competition with increasing molar excesses of the homologous AP-1#1 oligonucleotide (Fig. 3B, top panel, lanes 3 to 4). However, this complex was not competed for by the same molar excesses of the AP-1#1mut oligonucleotide (Fig. 3B, top panel, lanes 5 to 6), thereby demonstrating that selected mutations abolish AP-1 binding to the AP-1#1 site. Moreover, we confirmed the lack of AP-1 binding to the mutated AP-1#1mut probe and the absence of new complexes compared to the pattern observed with the wild-type probe (data not shown).

The same approach was followed to identify mutations hindering AP-1 binding to the AP-1#2 and AP-1#3 sites of fragment 5103 and to demonstrate their ability to abolish *in vitro* AP-1 binding to their respective sites (Figures 3A and 3B, respectively). Moreover, we confirmed the lack of AP-1 binding to the mutated AP-1#2mut and AP-1#3mut probes (data not shown).

In conclusion, we identified selected mutations which abrogate AP-1 binding to their respective binding site without altering the underlying amino acid sequence of the HIV-1 reverse transcriptase.

### The intragenic AP-1 binding sites are fully responsible for the PMA-dependent enhancer activity of fragment 5103

In order to address the potential functional role of the three intragenic AP-1 binding sites in the context of the whole 5103 fragment, we inserted this 264-bp fragment into the pTK reporter construct immediately upstream of the TK-luciferase transcriptional unit in the sense or antisense orientation, thereby generating the pTK-5103s-wt and the pTK-5103as-wt, respectively. The constructs pTK, pTK-5103s-wt and pTK-5103as-wt were transiently cotransfected into the human epithelial HeLa cell line with the pRL-TK (in which a cDNA encoding the *Renilla* luciferase is under the control of the HSV TK promoter region) used as an internal control for transfection efficiency. Twenty-four hours post-transfection, cells were mock-treated or treated with PMA. Twenty-four hours post-treatment, cells were lysed and assayed for luciferase activity.

**Figure 4:**
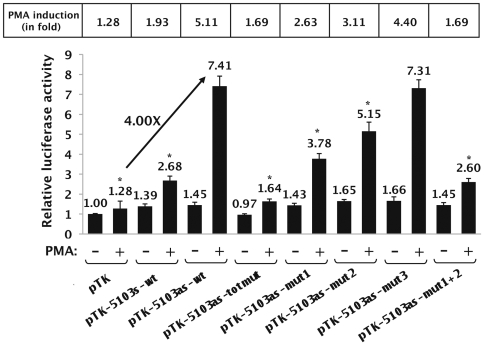
The intragenic AP-1 binding sites are fully responsible for the PMA-dependent enhancer activity of fragment 5103. HeLa cells were transiently transfected with 200 ng of the following constructs: the control vector pTK, the pTK-5103s-wt, pTK-5103as-wt, pTK-5103as-totmut, pTK-5103as-mut1, pTK-5103as-mut2, pTK-5103as-mut3 or pTK-5103as-mut1+2 reporter construct. All transfection mixtures additionally contained the pRL-TK (1 ng), in which a cDNA encoding the *Renilla* luciferase gene is under the control of the HSV TK promoter. At 24 h post-transfection, cells were mock-treated (-) or treated with PMA (+) (20 nM). Luciferase activities (*Firefly* and *Renilla*) were measured in cell lysates 48 h after transfection. Results are expressed as Luciferase*_Firefly_*/[Proteins]*Luciferase*_Renilla_* and presented as histograms indicating the luciferase activities of each construct relative to that of the control vector pTK, which was assigned an arbitrary value of 1 in absence of PMA. Means and standard errors of the means from three independent transfection experiments each performed in triplicate are indicated. The PMA induction of each TK construct is written in the upper part of the Figure (in fold). * indicates p<0.05 compared with the wild-type construct.

As shown in Figure 4, the plasmids pTK-5103s-wt and pTK-5103as-wt presented no significant activity compared to that of the control vector pTK in basal conditions. However, in the presence of PMA, transfection of plasmid pTK-5103s-wt or of plasmid pTK-5103as-wt resulted in a 1.93-fold ( = 2.68/1.39) or in a 5.11-fold ( = 7.41/1.45) induction of the measured luciferase activity, respectively. By normalizing the results in agreement with the control vector pTK activity (1.28-fold) following PMA-treatment of the cells, the observed increases in luciferase activity of these constructs represent a 1.51-fold ( = 1.93/1.28) or a 4.00-fold ( = 5.11/1.28) activation, respectively. These results indicate that the PMA-dependent enhancer activity of fragment 5103 shows a strong preference for the antisense orientation of this fragment with respect to the luciferase transcriptional unit.

We next investigated the relative contribution of each AP-1 binding site (AP-1#1, AP-1#2 and AP-1#3) to the PMA-dependent enhancer activity of fragment 5103. To this end, the mutations identified above as able to abolish AP-1 *in vitro* binding to their respective recognition sequence were introduced individually or in combination in the context of the pTK-5103as-wt by site-directed mutagenesis. The mutated plasmids were designated pTK-5103as-mut1, pTK-5103as-mut2, pTK-5103as-mut3, pTK-5103as-mut1+2 and pTK-5103as- totmut, and were tested for their PMA-responsiveness by transient transfection experiments in HeLa cells (Figure 4).

In basal conditions, the mutated constructs exhibited luciferase activities similar to that obtained with the control vector pTK, such as what we observed with the wild-type construct pTK-5103as-wt. Remarkably, following PMA-treatment of the cells, the plasmid pTK-5103as-totmut (where the three intragenic AP-1 sites have been mutated simultaneously) exhibited a luciferase activity similar to that obtained with the control vector pTK under the same conditions (Fig. 4), thereby indicating that the PMA-dependent enhancer activity of fragment 5103 required the integrity of the AP-1 motifs located in this fragment. In addition, transfection of plasmids pTK-5103as-mut1, pTK-5103as-mut2 and pTK-5103as-mut3 resulted in a 2.63-fold ( = 3.78/1.43), 3.11-fold and 4.40-fold PMA induction, respectively, compared to the 5.11-fold PMA induction obtained with the wild-type construct pTK-5103as-wt (Fig. 4). This corresponds to a 49% and 39% loss in the PMA-dependent enhancer activity of fragment 5103 when the AP-1#1 or the AP-1#2 site were mutated, respectively, whereas mutation in the AP-1#3 binding site resulted in a less pronounced loss of this activity (14% loss). Of note, the plasmid pTK-5103as-mut1+2 exhibited a PMA-responsiveness analogous to that obtained with the pTK-5103as-totmut (1.69-fold), suggesting that the two first AP-1 binding sites played a major role in the PMA-dependent enhancer activity of fragment 5103.

In conclusion, these results demonstrate that the PMA-dependent enhancer activity of fragment 5103 displays a strong preference for the antisense orientation of this fragment with respect to the transcriptional unit. Moreover, the loss of AP-1 binding to fragment 5103 significantly altered its PMA-dependent enhancer activity.

### The PMA-dependent enhancer activity of fragment 5103 is impaired by ectopically expressed dominant-negative A-Fos mutant

**Figure 5:**
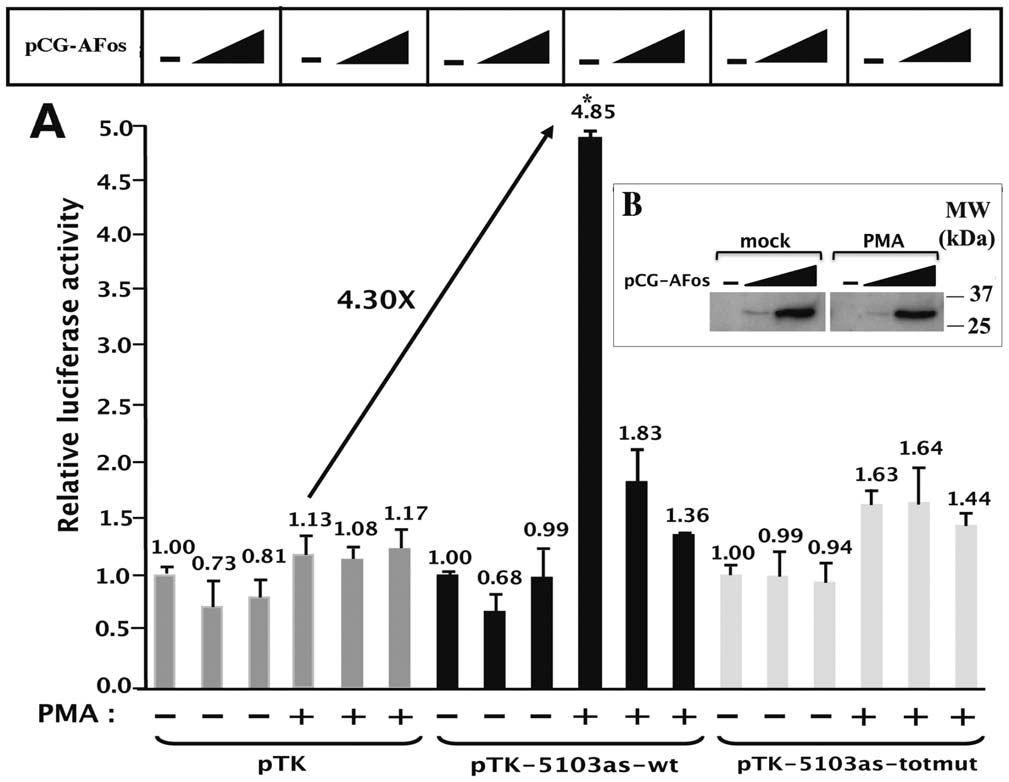
Overexpression of the dominant negative A-Fos mutant impairs the PMA-dependent enhancer activity of fragment 5103. (**A**) HeLa cells were transiently cotransfected with 100 ng of the pTK, pTK-5103as-wt or pTK-5103as-totmut reporter construct and with increasing amounts (0, 50 or 100 ng) of the dominant negative construct pCG-AFos. To maintain the same amount of transfected DNA and to avoid squelching artifacts, the different quantities of A-Fos expression vector cotransfected were complemented to 100 ng of DNA by using the empty vector pCG. Cells were additionally cotransfected with 1 ng of pRL-TK. Twenty-four hours post-transfection, cells were mock-treated (-) or treated with PMA (+). Luciferase activities (*Firefly* and *Renilla*) were measured in cell lysates 48 h after transfection. Results are expressed as Luciferase*_Firefly_*/Luciferase*_Renilla_* and presented as histograms indicating the relative luciferase activity of each construct with respect to the activity of the same reporter construct in the absence of both PMA and A-Fos, which was assigned an arbitrary value of 1. Means and standard errors of the means from three independent transfection experiments each performed in triplicate are indicated, with p<0.01 compared with the empty vector pTK indicated by an asterisk. (**B**) **A-Fos mutant expression is efficient and not affected by PMA-treatment of the cells.** HeLa cells were transiently transfected with increasing amounts (0, 2 or 4 µg) of the dominant negative construct pCG-AFos. To maintain the same amount of transfected DNA, the different quantities of A-Fos expression vector transfected were complemented to 4 µg of DNA by using the empty vector pCG. Twenty-four hours post-transfection, cells were mock-treated (mock) or treated with PMA (PMA) for one hour. Nuclear extracts were then prepared and analysed by western blot with an antibody directed against the N-terminal FLAG epitope of the A-Fos mutant.

To further confirm the crucial role played by AP-1 transcription factors in the PMA-dependent enhancer activity of fragment 5103, we evaluated the effects of overexpression of a dominant negative A-Fos mutant on this activity in transient transfection experiments. The dominant negative A-Fos mutant contains an acidic amphipathic protein sequence appended onto the N-terminus of the Fos leucine zipper, replacing the normal basic region critical for DNA binding [24]. This acidic extension and the Jun basic region form a heterodimeric coiled-coil structure that stabilizes the complex and prevents the basic region of Jun from binding to DNA, thereby titrating the functionally active AP-1 heterodimer concentration in the cell without altering the function of other B-ZIP proteins [24], [33]. We transiently cotransfected the control vector pTK, the pTK-5103as-wt or the pTK-5103as-totmut construct with increasing amounts of the expression vector encoding the A-Fos mutant (pCG-AFos). Twenty-four hours post-transfection, HeLa cells were mock-treated or treated with PMA. Twenty-four hours post-induction, cell lysates were assayed for luciferase activity (Figure 5A).

In agreement with the results presented in Figure 4, in the absence of A-Fos, transfection of the plasmid pTK-5103as-wt caused a 4.30-fold ( = 4.85/1.13) increase in luciferase activity following PMA-treatment of the cells (Figure 5A), whereas mutations present in pTK-5103as-totmut impeded the PMA-dependent enhancer activity of the 5103 fragment (Fig. 5A). Moreover, ectopic expression of A-Fos inhibited the PMA-dependent enhancer activity of fragment 5103 in a dose-dependent manner, while it did not affect the transcriptional activity of the control vector pTK (Fig. 5A). These results confirmed the important functional role of AP-1 transcription factors in the PMA-dependent enhancer activity of the 5103 fragment. Furthermore, as ectopic expression of A-Fos did not significantly affect the luciferase activity of the pTK-5103as-totmut construct neither in mock-treated nor in PMA-treated HeLa cells (Fig. 5A), we assumed that inhibition of the enhancer activity of fragment 5103 by A-Fos required intact intragenic AP-1 binding sites. Using the N-terminal FLAG epitope present on the dominant negative A-Fos mutant, we verified by western blot experiments that A-Fos was effectively expressed in a dose-dependent manner and that its production was not affected by PMA-treatment of the cells (see Figure 5B).

Altogether, these results demonstrate that AP-1-dimerizing proteins are the transcription factors fully responsible for the PMA-dependent enhancer activity of fragment 5103 through interaction with the intragenic AP-1 binding sites.

### The binding and functional properties of the AP-1 sites located in fragment 5103 are independent of HIV-1 Tat protein expression

HIV-1 transcription is boosted by the viral transactivating protein Tat, which binds to the *cis*-acting RNA TAR (Transactivation Response element) element located at the 5′end of all nascent viral transcripts in order to promote processive elongation [34]. In addition to viral transactivation, Tat has been shown to modulate the activity of several transcription factors either via direct interaction (such as previously reported for Oct-2 [35] and NFAT [36]) or via indirect mechanisms (including Tat-mediated alteration of Sp1 [37], NF-κB [38] and AP-1 [36] transcriptional activities). This prompted us to test whether Tat could alter the functional role of the intragenic AP-1 sites.

**Figure 6:**
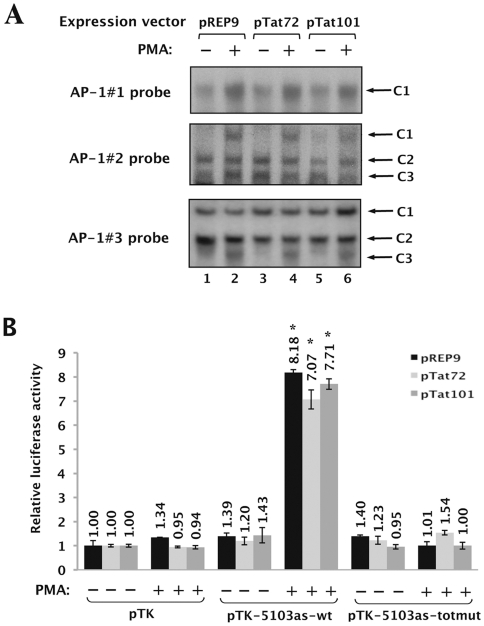
Binding and functional properties of intragenic AP-1 sites are independent of Tat protein expression. (**A**) HeLa cells were transiently transfected with the expression vector pTat72 encoding the one-exon form of Tat (72 amino acids named Tat72), the expression vector pTat101 encoding the two-exon form of Tat (101 amino acids or Tat101), or with the corresponding empty vector pREP9 used as a control. Twenty-four hours post-transfection, cells were mock-treated (-) or treated with PMA (+) for one hour. Nuclear extracts from these transiently transfected cells were prepared and used in EMSA experiments. The AP-1#1, AP-1#2 and AP-1#3 probes incubated with 10 µg of nuclear extracts from HeLa cells (lanes 1 and 2), HeLa cells expressing Tat72 (lanes 3 and 4) or HeLa cells expressing Tat101 (lanes 5 and 6), which were mock-treated (lanes 1, 3 and 5) or treated with PMA (lanes 2, 4 and 6). Retarded bands of interest are shown and indicated by arrows. (**B**) HeLa cells were transiently cotransfected with 100 ng of the pTK, pTK-5103as-wt or pTK-5103as-totmut reporter construct and with 200 ng of an expression vector encoding either the one-exon form of Tat (pTat72) or the two-exon form of Tat (pTat101) or of the corresponding empty vector pREP9. All transfection mixtures contained 1 ng of pRL-TK as a control of transfection efficiency. Twenty-four hours post-transfection, cells were mock-treated (-) or treated with PMA (+). Luciferase activities (*Firefly* and *Renilla*) were measured in cell lysates 48 h post-transfection. Results are expressed as Luciferase*_Firefly_*/[Proteins]*Luciferase*_Renilla_* and presented as histograms indicating the relative luciferase activities with respect to the activity of the control vector pTK in the absence of PMA, which was assigned an arbitrary value of 1. Means and standard errors of the means of one representative experiment from three independent transfection experiments each performed in triplicate are indicated. * indicates p<0.01 compared to the empty vector pTK.

To determine whether Tat expression affects the *in vitro* binding of AP-1 factors to each individual intragenic AP-1 binding site, we performed EMSAs with nuclear extracts from mock-treated or PMA-treated HeLa cells expressing the one-exon form of Tat (pTat72, see the Materials and Methods section), or the two-exon form of Tat (pTat101) (Figure 6A). Both forms of the Tat protein were studied because Tat101 is expressed both early and late in the virus life cycle, while Tat72 is only expressed in the late phase [39]. We observed no significant differences when we compared the retarded complexes in absence of Tat versus in presence of Tat (Figure 6A, compare lane 1 to lanes 3 and 5; lane 2 to lanes 4 and 6), neither in basal nor in PMA-induced conditions. Tat expression in these nuclear extracts was verified by ELISA (data not shown). These results show that Tat expression does not modulate the *in vitro* binding of AP-1 to fragment 5103.

We next evaluated the potential impact of Tat expression on the PMA-dependent enhancer activity of fragment 5103. Therefore, we transiently cotransfected HeLa cells with the control vector pTK, the pTK-5103as-wt or the pTK-5103as-totmut constructs and with an expression vector encoding the Tat protein (pTat72, pTat101 or the corresponding empty vector pREP9). Twenty-four hours post-transfection, cells were mock-treated or treated with PMA. Twenty-four hours post-induction, cells were lysed and assayed for luciferase activity.

As shown in Figure 6B, the PMA-dependent enhancer activity of fragment 5103 observed in the absence of Tat (a 6.11-fold increase in luciferase activity following PMA-treatment of the cells with the pTK-5103as-wt construct compared to the activity of the pTK) was not affected neither by Tat72 expression nor by Tat101 expression (Fig. 6B; increasing concentrations of the Tat expression vectors were used but only the highest dose is shown). Control experiments confirmed that Tat was expressed and was functional in terms of transactivation of the HIV-1 promoter in our experimental conditions (data not shown).

In conclusion, these results indicate that the *in vitro* binding of AP-1 factors to the three intragenic AP-1 sites and the functional role of these sites in the PMA-dependent enhancer activity of fragment 5103 are independent of Tat protein expression.

### Mutations in the intragenic AP-1 binding sites impair c-Fos, JunB and JunD *in vivo* recruitment to the 5103 fragment and lead to a decreased recruitment of RNA polymerase II to the viral promoter

In order to determine whether endogenous AP-1 transcription factors are recruited *in vivo* to the HIV-1 5103 fragment, we conducted chromatin immunoprecipitation (ChIP) experiments. HeLa cells were transiently transfected with the wild-type plasmid pHIV-1 (HIV-1 full-length molecular clone pNL4-3) or with its mutated counterpart pHIV-1-AP-1#totmut (mutated in the three intragenic AP-1 sites simultaneously). Twenty-four hours post-transfection, cells were mock-treated or treated with PMA. Twenty-four hours post-induction, chromatin was prepared from these cells and immunoprecipitated with specific antibodies directed against c-Fos, JunB, JunD, Fra-1, RNA polymerase II (RNAPII) or with a purified IgG antibody as a control. Three different primer pairs were designed in the 5103 fragment region of interest, in the nuc-1 region where AP-1 binding sites had been previously reported [8], [9] and in the *vpr* gene, where neither the literature nor our *in silico* analyses have revealed the presence of potential AP-1 binding sites (data not shown).

**Figure 7:**
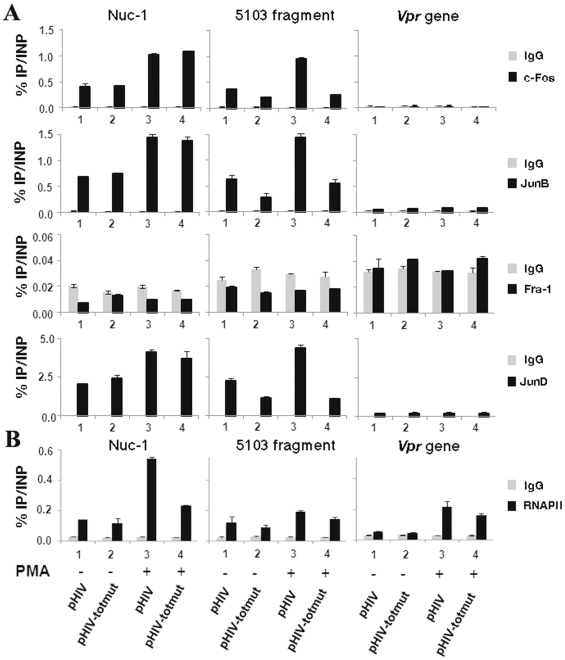
The AP-1 transcription factors c-Fos, JunB and JunD are recruited *in vivo* to the 5103 fragment region. HeLa cells were transiently transfected with the wild-type pHIV-1 or the mutated pHIV-1-AP-1#totmut construct. Twenty-four hours post-transfection, cells were mock-treated (-) or treated with PMA (+). Twenty-four hours post-induction, cells were cross-linked for 10 min at room temperature with 1% formaldehyde. To detect chromosomal flanking regions, pellets were sonicated to obtain DNA fragments of an average size of 400 bp. Chromatin immunoprecipitations were performed with specific antibodies directed against c-Fos, JunB, Fra-1 or JunD. To test aspecific binding to the beads, a purified IgG was used as a control for immunoprecipitation. Quantitative PCR reactions were performed with oligonucleotide primers hybridizing either in the nuc-1 region (termed nuc-1), or in a region overlapping the three AP-1 binding sites of the 5103 fragment (termed 5103 fragment), or in the *vpr* gene (termed *vpr* gene) where no AP-1 binding sites have been previously reported. Fold enrichments were calculated as percentages of immunoprecipitated DNA following the formula “Immunoprecipitated DNA (IP)*100/Input DNA (INP)”. Values represent the means of triplicate samples and standard errors of the means are indicated. An experiment representative of three independent ChIP assays is shown. (**B**) **Mutations in the intragenic AP-1 sites affect the PMA-inducible** ***in vivo*** **recruitment of RNA polymerase II to the HIV-1 5′LTR region.** Chromatin immunoprecipitations were performed with a specific antibody directed against RNAPII and a purified IgG as a control. Quantitative PCR reactions were performed with the same oligonucleotide primers and fold enrichments were calculated as in panel (A). Means and standard errors of the means from one experiment representative of three independent ChIP assays are shown.

As shown in Figure 7A (central panels), we observed the *in vivo* recruitment of c-Fos, JunB and JunD, but not of Fra-1, to the 5103 fragment region. This recruitment increased following PMA-treatment of the cells (compare lanes 1 and 3). These results are in good agreement with our *in vitro* binding studies (see above Figure 2B). Importantly, AP-1 recruitment to the 5103 fragment region was significantly affected by mutations introduced in the three intragenic AP-1 binding sites of the pHIV-1#totmut construct, both in absence (Fig. 7A; compare lanes 1 and 2) and in presence (compare lanes 3 and 4) of PMA. Interestingly, c-Fos, JunB and JunD (but not Fra-1) were shown to be recruited to the nuc-1 region (Fig. 7A, left panels), where they had been previously demonstrated to bind (or not to bind concerning Fra-1) *in vitro* to the AP-1 binding sites identified in the 5′LTR [40]. As a control, no binding of AP-1 family members was observed in the HIV-1 *vpr* gene region (see Fig. 7A, right panels).

Moreover, the *in vivo* recruitment of RNA polymerase II to the 5′LTR region increased following PMA treatment of the cells (Figure 7B; left panel). Remarkably, the combined mutation of the three intragenic AP-1 binding sites decreased by ∼ two fold the PMA-mediated RNAPII *in vivo* recruitment to the viral promoter (Fig. 7B, left panel), thereby supporting the notion that the 5103 fragment AP-1 binding sites are important for HIV-1 transcriptional activity.

Altogether, these results indicate that c-Fos, JunB and JunD (but not Fra-1) are recruited *in vivo* to the 5103 fragment region in a PMA-inducible manner in the context of our transient transfection experiments. They also confirm that the designed mutations affect c-Fos, JunB and JunD *in vivo* recruitment to the 5103 fragment region without altering AP-1 recruitment to the nuc-1 region. Importantly, mutations of the three intragenic AP-1 sites decrease RNAPII *in vivo* recruitment to the viral promoter, supporting the notion that these sites are important for HIV-1 transcriptional activity.

### The intragenic AP-1 binding sites are important for viral replication

**Figure 8:**
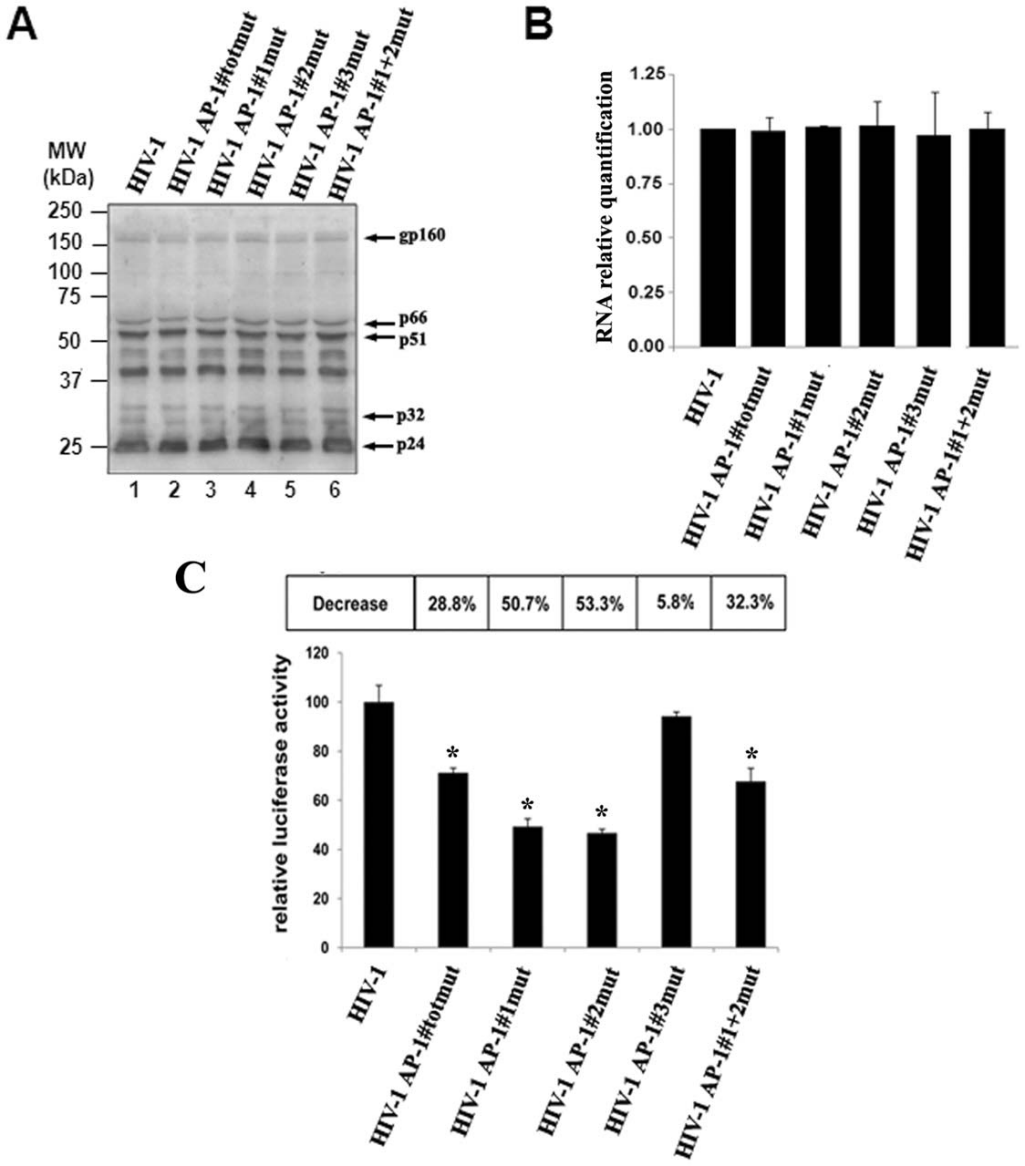
Viral particles from mutant and wild-type virus stocks are similar at the protein and at the RNA levels. (A) Equivalent amounts of viral particles (as assessed by p24 ELISA assays) from the wild-type and each AP-1 mutant virus stocks were pelleted by centrifugation, lysed in Laemmli buffer and analyzed by Western blotting with an anti-HIV-1 immunoglobulin. The bands corresponding to the HIV-1 glycoprotein gp160, reverse transcriptase p66/p51, integrase p32 and capsid p24 proteins are indicated. MW, molecular weight (indicated in kDa). (B) Viral RNAs from equal amounts of viral particles were digested with DNase I and subsequently reverse-transcribed with random primers. First-strand viral cDNAs were then quantified by qPCR with primers hybridyzing in the TAR region (as described in the Materials and Methods section). An arbitrary value of 1 was assigned to the result obtained with the wild type virus stock HIV-1. Means and standard errors of the means from two independent experiments each performed in triplicate are indicated. (**C**) **Mutations in the intragenic AP-1 binding sites affect HIV-1 expression.** TZM-bl cells (6×10^3^ cells) were infected or not with equal amounts of wild-type HIV-1 or mutant virus stocks. At 72 h post-infection, TZM-bl cells were lysed and luciferase activity was measured in cell lysates. Results are presented as histograms indicating the Luciferase*_Firefly_* activity of the TZM-bl cells following infection with wild-type versus mutant viruses. An arbitrary value of 100% was attributed to the result obtained with the wild-type HIV-1. Means and standard errors of the means from one representative from three independent experiments each performed in triplicate are indicated. * indicates p<0.05 compared to the wild-type virus HIV-1.

We next studied the biological significance of the 5103 fragment AP-1 binding sites for HIV-1 replication. Stocks of wild-type and mutant HIV-1 infectious viruses were produced as described in the Materials and Methods section and used in infection assays. In order to check that the potential effects of introduced mutations on HIV-1 replication do not result from a serious defect in HIV-1 protein content or in HIV-1 RNA genome packaging of the virus stocks used to perform infectivity studies, lysates and viral RNA from equal amounts of p24 from the wild-type and mutant virus stocks were respectively analysed by western blotting with a purified human anti-HIV-1 IgG antibody and by RT-qPCR to quantify the amount of viral genomic RNA in each virus stock (Figures 8A and 8B, respectively). Similar amounts of each HIV-1 detected proteins, including the reverse transcriptase which is partially encoded by fragment 5103, and of viral genomic RNA were observed in all samples, supporting the notion that all virus stocks used in the infection studies were structurally similar at both the protein and RNA levels.

In order to evaluate the importance of the intragenic AP-1 sites for viral expression, we infected TZM-bl cells with the wild-type (HIV-1) and mutant (HIV-1-AP-1totmut; HIV-1-AP-1#1mut, HIV-1-AP-1#2mut, HIV-1-AP-1#3mut and HIV-1-AP-1#1+2mut) viral stocks (Figure 8C). TZM-bl cells, that contain the luciferase gene under the control of the HIV-1 LTR, are easily infected by HIV-1 since they express the receptor CD4 and both co-receptors CXCR4 and CCR5. Once integrated into the cellular genome, the virus begins expressing the viral Tat protein, which then activates luciferase expression by trans-activating the LTR transcriptional activity. Thus, luciferase expression in those cells is a reliable reporter for viral expression. As shown in Figure 8C, mutations in the intragenic AP-1 binding sites affected luciferase expression compared to the wild-type HIV-1, especially mutation in the first and second AP-1 sites which caused a decrease of luciferase expression of 50.7% and 53.3%, respectively.

Altogether, these results show that mutations in the 5103 fragment AP-1 sites affect HIV-1 expression. These deleterious effects observed with mutant viral stocks are likely to be a direct consequence of impaired AP-1 binding to the mutated 5103 fragment since wild-type and mutant HIV-1 particles from the viral stocks used in the infection studies are structurally similar at both the RNA and protein levels.

### Mutations in the intragenic AP-1 sites alter HIV-1 replication in T-lymphoid and promonocytic cell lines

**Figure 9:**
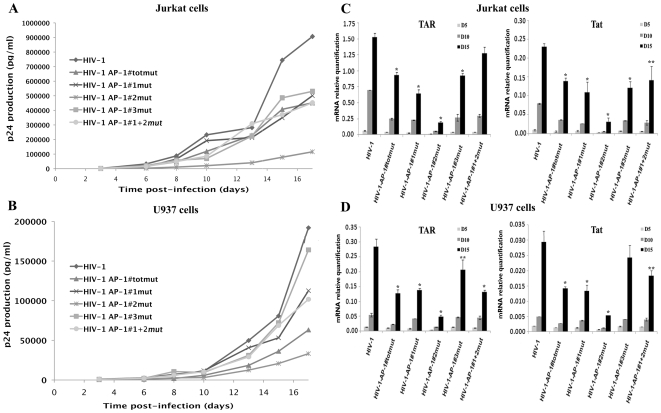
The AP-1 binding sites located in the 5103 fragment are important for viral replication. Jurkat (A and C) or U937 (B and D) cells were infected with equivalent amounts of p24 concentration of wild-type (HIV-1), totally mutated (HIV-1-AP-1totmut), partially mutated (HIV-1-AP-1#1+2mut) or individually mutated (HIV-1-AP-1#1mut, HIV-1-AP-1#2mut and HIV-1-AP-1#3mut) viral infectious stocks as described in the Materials and Methods section. (A and B) Viral production in cell supernatants was quantified by measuring p24 antigen concentration in the culture supernatants at different times following infection. The experiment shown is representative of at least 3 independent infection experiments (each performed in triplicate). The means are presented and the variation for a given mutant between different experiments was <15% in each case. (C and D) Total RNA was extracted from infected cells at days 5, 10 and 15 post-infection, digested with DNase I and reverse-transcribed using random primers. First-strand cDNAs were analyzed by qPCR with the comparative C_t_ (ΔΔC_t_) quantification method using the two following sets of primers: initiated transcripts (TAR primers) and elongated transcripts (Tat primers) were quantified using β-actin to normalize the results. Means and standard errors of the means from two independent experiments each performed in triplicate are indicated. * and ** indicate p<0.05 and p<0.1 compared to the wild-type virus HIV-1 at day 15 post-infection.

In order to address the importance of the 5103 fragment AP-1 binding sites for HIV-1 replication in the two major cell targets of HIV-1, i.e. T cells and macrophages, we infected T-lymphoid Jurkat and promonocytic U937 cells with the wild-type and mutant viral stocks. We subsequently monitored the growth kinetics of the infection by measuring p24 production in the cell supernatants over a period of 17 days and by quantifying viral mRNA production at days 5, 10 and 15 post-infection. Figures 9A and 9B show a representative replication curve of three independent replication assays (each performed in triplicate) for Jurkat and U937 cells, respectively.

Infection of T-lymphoid Jurkat cells with the wild-type virus resulted in a strong viral production (Fig. 9A), whereas mutant viruses revealed differences in replication rate and/or virus production levels compared to the wild-type virus. At day 20 and later on, a rapid decrease in p24 production was observed, reflecting the heavy reduction of viable cell counts (data not shown). Mutant viruses HIV-1-AP-1#3mut, HIV-1-AP-1#1mut, HIV-1-AP-1#1+2mut and HIV-1-AP-1#totmut exhibited replication kinetics analogous to that observed with the wild-type control virus HIV-1, but with lower levels of viral production (corresponding to a 46%, 49%, 53% and 53% decrease, respectively, of p24 release from infected cultures on day 17 compared to the p24 release observed with the wild-type HIV-1) (Fig. 9A), thereby indicating a deleterious effect of the introduced mutations for HIV-1 viral production. Moreover, infection of Jurkat cells with the HIV-1-AP-1#2mut virus led to a very low p24 release (reaching 88% decrease on day 17 compared to the wild-type virus), reflecting severely reduced replication properties (Fig. 9A). As shown in Figure 9B, all viruses produced lower concentrations of viral p24 in promonocytic U937 cells and mutant viruses affected HIV-1 replication in a similar manner than what was observed in T-lymphoid Jurkat cells, except for mutant HIV-1-AP-1#3mut. Of note, the HIV-1-AP-1#2mut virus exhibited severely reduced replication properties, reaching 83% decrease of the p24 production at day 17. A second round of infection of target Jurkat and U937 cells with supernatants collected 18 days after the first infection allowed us to conclude that all mutant viruses were competent in terms of infectivity in both cell lines (data not shown).

In parallel, we quantified initiated (TAR) and elongated (Tat) viral mRNAs produced in Jurkat and U937 infected cells at days 5, 10 and 15 post-infection (see Figures 9C and 9D, respectively). We observed similar patterns of viral production with mutant versus wild-type viral stocks than those observed by measuring p24 production in cell supernatants. Indeed, mutant HIV-1-AP-1#2mut exhibited a highly affected replication profile in both cell lines (reaching a 87% and 82% decrease of elongated transcripts in Jurkat and U937 cells at day 15 post-infection, respectively; Figures 9C and 9D). Mutant viruses HIV-1-AP-1#3mut, HIV-1-AP-1#1mut, HIV-1-AP-1#1+2mut and HIV-1-AP-1#totmut exhibited a similar behaviour than previously measured in p24 assays since we observed a 48%, 52%, 39% and 40% (17%, 54%, 37% and 52%) decrease of elongated transcripts in Jurkat (U937) cells at day 15 post-infection.

The HIV-1-AP-1#totmut and the HIV-1-AP-1#1+2mut viruses were less affected than the virus containing the individual AP-1#2 site mutation, suggesting partial compensatory mechanisms turned on by the virus when two or three intragenic AP-1 sites are mutated simultaneously to ensure its replication. These mechanisms could, for example, involve the recruitment of other transcription factors or co-factors in the intragenic region.

**Figure 10:**
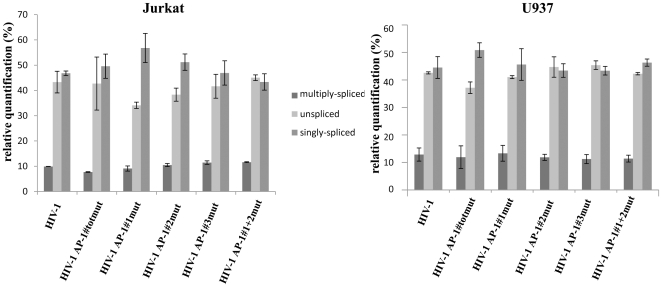
The splicing pattern of HIV-1 transcripts is unaffected by the mutations introduced in the intragenic AP-1 binding sites. Total RNA was extracted from infected Jurkat or U937 cells (with wild-type or mutant virus stocks) at 5 days post-infection. After DNase I treatment, total RNA was reverse-transcribed with random primers and first strand cDNAs were quantified with primers designed to quantify full-length unspliced viral mRNAs, singly-spliced viral mRNAs and multiply-spliced viral mRNAs. The total amount of viral mRNAs for each virus was arbitrarily attributed a value of 100% and the proportion of each type of transcript is presented as histograms indicating the means and standard errors of the means from two independent experiments performed in duplicate.

Noteworthy, we verified that mutations introduced in the intragenic AP-1 sites did not significantly modify the splicing pattern of viral transcripts by quantifying unspliced full-length (9 kb), singly-spliced (4 kb) and multiply-spliced (2 kb) transcripts for each mutant virus after infection (see Figure 10). We also demonstrated that the reduced replication phenotypes observed with the AP-1 mutant viruses were not due to a defect neither in the protein nor in the RNA content of viral particles (Figures 8A and 8B, respectively). Moreover, our ChIP results showed that the role of intragenic AP-1 sites in HIV-1 replication takes place, at least partly, at the transcriptional level since the increased RNAPII *in vivo* recruitment to the viral promoter following PMA treatment of the cells was affected by mutations in the intragenic AP-1 binding sites (Figure 7B).

Taken together, our results demonstrate that the integrity of the 5103 fragment AP-1 binding sites located 4 kb downstream of the HIV-1 transcription start site is important, notably at the transcriptional level, for an efficient HIV-1 replication in human CD4^+^ T-lymphocytes and promonocytic cell lines, indicating a positive regulatory function of the intragenic AP-1 sites.

### Single-round HIV-1 infections of MDMs are affected by mutations in the *pol* gene AP-1 binding sites

**Figure 11:**
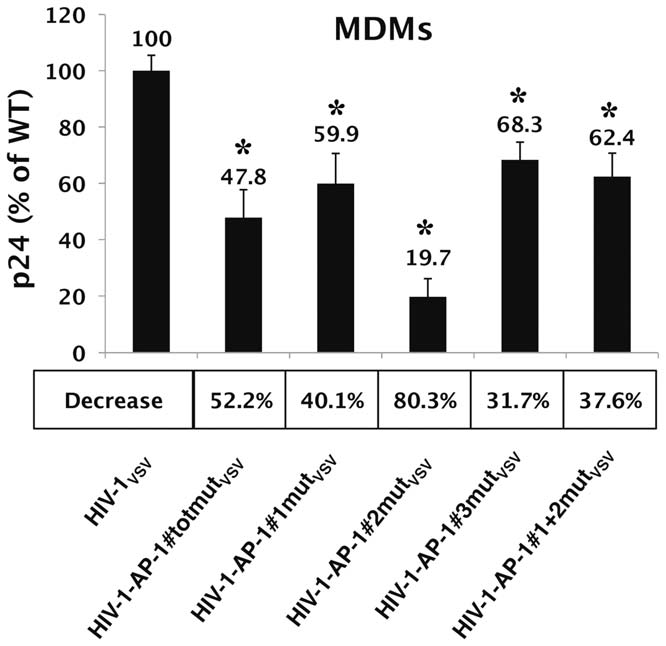
Single-round HIV-1 infections of MDMs are affected by mutations in the intragenic AP-1 binding sites. Monocyte-derived macrophages from three healthy donors were isolated and individually infected with VSV-G pseudotyped HIV-1_NL4.3_ viruses (HIV-1_VSV_, HIV-1-AP-1#totmut_VSV_, HIV-1-AP-1#1mut_VSV_, HIV-1-AP-1#2mut_VSV_, HIV-1-AP-1#3mut_VSV_, HIV-1-AP-1#1+2mut_VSV_). Production of p24 in the culture supernatant was measured by ELISA at day 10 post-infection. Results are presented as histograms indicating the p24 production level of each mutant virus compared to the wild-type virus HIV-1_VSV_, which was assigned an arbitrary value of 100%, and correspond to results obtained from three independent donors in order to take into account the variability that may exist between donors. Means and standard errors of the means are indicated. * indicates p<0.05 compared to the wild-type virus HIV-1_VSV_.

AP-1 transcription factors are known to play an important role in myeloid cells [41]. Moreover, the DNaseI-hypersensitive site HS7, which is part of the intragenic region, has been identified in a cell line of monocytic origin (U1), whereas it was absent in two cell lines of lymphoid origin (ACH2 and 8E5) [3], [5]. Therefore, we further evaluated the importance of the three AP-1 binding sites of fragment 5103 on HIV-1 replication in primary monocyte-derived macrophages (MDMs). Since HIV-1_NL4.3_ strain is not able to infect human MDMs [42], we used wild-type and mutant HIV-1 viruses pseudotyped with the VSV-G glycoprotein to assess the impact of the AP-1 mutations on viral expression in single-round infections. p24 production in infected cell supernatants was used as a marker of viral expression. Results of experiments performed with MDMs from 3 different donors are presented as percentages of p24 levels at day 10 for each mutant virus compared to the wild-type virus (Figure 11).

p24 production resulting from MDMs transduction by the mutants HIV-1-AP-1#1mut_VSV_, HIV-1-AP-3#totmut_VSV_, HIV-1-AP-1#1+2mut_VSV_ and HIV-1-AP-1#totmut_VSV_ viruses was reduced compared to that observed with the wild-type HIV-1_VSV_ (leading to a 40.1%, 31.7%, 37.6% and 52.2% decrease, respectively). Importantly, mutation in the AP-1#2 site strongly impaired MDMs transduction (80.3% decrease in p24 production compared to the wild-type). Interestingly, mutation in the second AP-1 site was the most deleterious in both cell lines and primary macrophages, indicating an important role of the AP-1#-2 site for HIV-1 replication. PCR quantification of late reverse transcriptase products did not show substantial reductions in MDMs infected with AP-1 mutated viruses compared to MDMs infected with the wild-type HIV-1 virus, which could have explained the reduction in p24 production we observed in our infection studies with mutated viruses compared to wild-type virus (data not shown). Therefore, these results suggest that the defect in viral expression in MDMs transduced by the AP-1 mutated viruses is not caused by pre-integration blocks.

Altogether, these results demonstrate the importance of the intragenic AP-1 binding sites for efficient HIV-1 replication in macrophages as a decrease in viral expression of AP-1 site mutant viruses, and especially of the AP-1#2 site mutant virus, was detected in transduction of primary MDMs with VSV-G pseudotyped viruses (during single-round infections).

## Discussion

Our laboratory has previously identified an important intragenic region in the HIV-1 genome, whose complete functional unit is composed of the 5103 fragment, the hypersensitive site HS7 and the 5105 fragment [2], [3], [5], [6] (Figure 1). These two fragments (5103 and 5105) both exhibit a PMA-inducible enhancer activity on the HSV TK promoter in HeLa cells, but no significant activity in T-lymphoid and monocyte-macrophage cell lines [2]. AP-1 transcription factors are typically absent in quiescent cells but significantly induced upon cellular activation, notably following phorbol ester treatment of the cells [43]. In this regard, the three AP-1 binding sites of fragment 5103 [7] were good candidates to investigate the PMA-dependent enhancer activity of this fragment.

In the present report, we characterized biochemically and functionally these three intragenic AP-1 binding sites by showing the PMA-inducible *in vitro* binding and *in vivo* recruitment of the AP-1 family members c-Fos, JunB and JunD to the 5103 fragment. Our *ex vivo* transient transfection assays in the heterologous context of the HSV TK promoter demonstrated that the intragenic AP-1 sites are fully responsible for the PMA-dependent enhancer activity of fragment 5103. We further demonstrated that this activity was completely inhibited by the overexpression of a dominant-negative A-Fos mutant. Moreover, we investigated the biological significance of the intragenic AP-1 sites for HIV-1 replication. We produced wild-type and mutant viral stocks and demonstrated the importance of the 5103 fragment AP-1 sites for viral expression in HeLa-derived TZM-bl cells. Importantly, infection of T-lymphoid Jurkat and promonocytic U937 cell lines with wild-type and mutant viruses showed that mutation of the intragenic AP-1 sites individually or in combination altered HIV-1 replication. Our ChIP results demonstrated that the deleterious effect observed on HIV-1 replication with mutant viruses occurs (at least partly) at the transcriptional level since we measured a ∼ two fold decrease of the PMA-mediated RNAPII *in vivo* recruitment to the viral promoter when the three intragenic AP-1 sites were mutated simultaneously. Remarkably, mutations in the *pol* gene AP-1 sites also affected viral replication in MDMs in single-round infection experiments, in agreement with the important role played by AP-1 in myeloid cells. Interestingly, at day 15 post-infection with the AP-1 mutant viruses, we observed the emergence of a low percentage (5 to 10%) of viruses that reverted to the wild-type sequence or that harboured additional mutations around the AP-1 sites possibly to counteract the negative effects of the intragenic AP-1 sites mutations on viral replication.

In this study, we observed some divergences between *ex vivo* transient transfection assays and *in vivo* infection experiments regarding the effects of certain AP-1 mutants. Several phenomena may explain these differences. They may result from the fact that our studies were performed in the heterologous context of the HSV TK promoter (for transfection assays) and in the homologous context of the entire HIV-1_NL4.3_ isolate (for infection experiments). Indeed, viral replication involves other transcription factors that bind elsewhere in the HIV-1 provirus, *cis*-regulating elements adjacent to fragment 5103 and the chromatin organization of the integrated provirus, notably of the intragenic region. In this regard, transient transfection experiments may not reflect the regulation found *in vivo* since transiently transfected DNA is not assembled into physiological chromatin. In addition, the pool of cellular transcription factors differs from one cell type to another, the differences in the availability of AP-1 (or of certain members of the AP-1 family) or of another co-factor important for AP-1 activity in those particular cell lines may have consequences on the observed effect of the intragenic AP-1 binding sites mutations for HIV-1 replication and/or for the 5103 fragment enhancer activity. Similar divergences between transient transfection studies and *in vivo* functional studies have been previously reported for HIV-1 by different groups including our laboratory [5], [6], [9], [44], [45], [46]. Moreover, it may seem surprising that the HIV-1 AP-1#2 mutant virus was more deleterious for HIV-1 replication than the HIV-1#1+2mut and the HIV-1#totmut viruses, which also contain the AP-1#2 site mutation. However, since HIV-1 suffers from a high error rate during the reverse transcription step, it has developed rescue mechanisms. Here, the virus may activate such rescue mechanisms when two or three AP-1 sites are defective, by recruiting other transcription factors or co-factors for instance, while mutation of a unique intragenic AP-1 site may not be sufficient to turn on these rescue mechanisms.

AP-1 transcription factors are known to play important roles in myeloid development [41], whereas they are slightly expressed in Jurkat cells [47]. The binding and functional studies reported here were performed in epithelial HeLa cells, which contain a high endogenous level of AP-1 transcription factors [47], because we could not observe PMA-mediated activation of AP-1 in the two other cell lines used (Jurkat and U937) despite many attempts (data not shown). Indeed, it has been reported that transformed cells often have demonstrable defects in cell signalling. For instance, Ras-mediated extracellular regulated kinase (ERK) activation varies between primary T cells and Jurkat T cells [48], [49]. Wabnitz and colleagues have reported that, whereas it is not the case in Jurkat lymphoma cells, in peripheral blood T lymphocytes, phorbol esters activate Ras and the phosphatidylinositol-3-kinase (PI3K) substrate Akt [50].

Importantly, our single-round infections of MDMs strongly supported the importance of the intragenic AP-1 sites for an efficient HIV-1 replication in macrophages since their mutation affected viral expression in transduced cells. Interestingly, the hypersensitive site HS7 has been identified in the latently-infected monocytic (U1) cell line, but not in the T-lymphoid (ACH2 and 8E5) cell lines [3], supporting a cell-type specific role of the intragenic region. Such cell-type-specific properties could result from the presence of several binding sites for other transcription factors, such as binding sites for the macrophage and B-cell specific transcription factor PU.1 that we identified in the intragenic region ([6] and unpublished data from our laboratory). Indeed, previous studies have reported that AP-1 and PU.1 can cooperate in the regulation of cellular genes in macrophages [48], such as for the adipose differentiation-related protein gene [51]. Interestingly, c-Jun homodimers can function as coactivators for PU.1 in macrophages as demonstrated on the monocyte-specific macrophage colony-stimulating factor (M-CSF) receptor promoter [52]. Further investigations concerning the functional interplay between the AP-1 and PU.1 binding sites in the intragenic region will be needed to unveil possible mechanisms involved in the cell-type-specific role of the intragenic region in macrophages. Other viruses have been shown to possess such intragenic regulatory regions contributing to transcriptional regulation by adding a cellular specificity, including the human hepatitis B virus and the closely related woodchuck hepatitis virus [53], [54].

Previous studies about the role played by AP-1 in HIV-1 transcription and expression were centred on the AP-1 binding sites present in the 5′LTR and leader region of HIV-1. TRE elements have been previously characterized in the NRE region (at positions nt 151-175 and nt 213-233) of different neuronal strains [8] and downstream of the transcription start site in the HS4 region [the AP-1(I) site (nt 541 to 547), AP-1(II) site (nt 572 to 578) and AP-1(III) site (nt 609 to 614)] [9]. Other viruses including feline immunodeficiency virus [55], foamy viruses [56], [57], Kaposi's sarcoma associated herpes virus [58], [59], human papillomavirus [60], [61], visna virus [62] or human T-cell leukemia/lymphoma virus type I (HTLV-I) [63], [64] use AP-1 factors either to regulate their own replication or to interfere with host cell gene regulation. In recent years, the interest for the role played by chronic immune activation and inflammation in HIV-1 pathogenesis increasingly raised. Indeed, quite paradoxically, chronic immune activation has been associated with high levels of viremia and is a primary driver of HIV-1 progression to AIDS. Of note, inducible transcription factors such as AP-1 or NF-κB are involved in inflammation processes and activate transcription of antiviral and inflammatory genes. They also increase HIV-1 expression level by binding to the viral 5′LTR and leader region and, concerning AP-1, in the intragenic *cis*-regulatory region. In this context, AP-1 proteins, among other inducible cellular transcription factors, might play a role both in the transactivation of viral gene expression and in the inflammation process that together lead to chronic immune activation in HIV-infected patients.

In conclusion, the 5103 fragment, containing the three intragenic AP-1 binding sites characterized in this report, in cooperation with the adjacent HS7 region and the 5105 fragment (Fig. 1) are the components of a large intragenic regulatory region that could either bring an additional cellular specificity, and/or increase the strength of the promoter/enhancer unit located in the HIV-1 5′LTR, and/or allow viral responses to a broader variety of exogenous stimuli. The intragenic AP-1 binding sites correspond to an additional factor in an already complex network of regulators affecting HIV-1 replication at the transcriptional level, thereby contributing to an efficient viral control of the infection.
